# Green nephrology in practice: actions that promote environmental, social, and economic impact

**DOI:** 10.1590/2175-8239-JBN-2024-0206en

**Published:** 2025-06-13

**Authors:** Fabiana Baggio Nerbass, Rosenilde Schulz, Maicon Aurélio de Vargas, Maycon Truppel Machado, Marcos Alexandre Vieira

**Affiliations:** 1 Fundação Pró-Rim, Joinville, SC, Brazil.

**Keywords:** Climate change, Dialysis, Recycling, Renewable Energy

## Abstract

Climate change affects - and will continue to affect - the overall and kidney health of millions of people worldwide. While healthcare services should be prepared for the increased incidence and changes in the distribution of kidney disease, they also substantially contribute to the depletion of natural resources and greenhouse gas emissions. Among these services, the dialysis sector stands out for its negative environmental impact, due to its high consumption of water, energy, and supplies, in addition to the waste it generates. Thus, measures that reduce the use of natural resources in dialysis procedures, as well as those aimed at reducing the number of patients dependent on dialysis, such as initiatives to prevent kidney disease and encourage kidney transplantation, are of paramount importance. In addition to environmental gains, internal actions focused on the implementation of sustainable practices may also generate economic returns. In this article, our objective was to inform and contribute to the knowledge on sustainable experiences already implemented in a Brazilian philanthropic healthcare institution. Among these, we describe how the use of renewable energy, the reuse of reverse osmosis reject water, and waste recycling translate into reduced operating costs and direct support for socially vulnerable patients.

## Introduction

According to the World Health Organization, climate change already contributes directly to humanitarian emergencies, and populations living in tropical areas, especially those in regions with poor healthcare infrastructure, will continue to experience the most severe consequences^1^.

In particular, kidney disease and climate change have a two-way relationship: each exacerbates the other^2^. A meta-analysis of 82 epidemiological studies found that for every 1°C rise in temperature, there was a 1% increase in morbidity and a 3% increase in mortality from kidney-related causes. In addition, heat waves were found to increase the risk of kidney disease proportionally to their intensity^3^.

Conversely, the treatment of chronic kidney disease, especially using hemodialysis (HD), has one of the highest ecological footprints across the entire spectrum of clinical care due to its high consumption of energy, water, and disposable plastic^4^, in addition to the generation of moderately toxic effluents^5^. HD is the dialysis modality used by approximately 90% of dialysis-dependent patients worldwide^6^ and by more than 95% of those undergoing treatment in Brazil, a figure that exceeds 150,000 individuals^7^.

In a recent publication, the “GREEN-K” initiative was announced: Global Environmental Evolution in Nephrology and Kidney Care, with the vision of “sustainable kidney care for a healthy planet and healthy kidneys”. Among other proposals, the initiative advocates for prioritizing health promotion actions to prevent and delay the progression of kidney disease, improve access to transplants, and address the urgent need for dialysis therapies with lower environmental impact^2^. A survey involving more than 972 healthcare professionals from 108 countries, focusing on the impacts of climate and environmental change on health and kidney care, revealed that although the vast majority expressed concern about these issues, only 26% reported working in organizations with sustainable initiatives. This percentage did not vary according to the country’s income level^8^.

A call for sustainability in dialysis in Brazil was published in 2019, but, as the article points out, the topic is still rarely discussed in the country. The authors highlight the need to promote and encourage the adoption of sustainability in dialysis centers nationwide, fostering the development of collective awareness^9^.

In addition to environmental gains, implementing sustainable practices could generate both economic and social benefits. In this article, our main objective is to share successful sustainable actions implemented by dialysis units belonging to a philanthropic institution, such as the use of renewable energy, the reuse of reverse osmosis reject water, and the recycling of materials, all of which translate into reduced operational costs and direct support to socially vulnerable patients.

## Institutional Environmental Management Policy

The institution is a private, philanthropic, non-profit organization founded in 1987, with a presence in the states of Santa Catarina and Tocantins, where it operates dialysis centers both independently and in partnership.

The institution’s environmental management policy was implemented shortly after the publication of RDC No. 306 of 2004, which establishes the Technical Regulations for the Health Services Waste Management Plan (PGRSS, for its acronym in Portuguese)^10^. The commitment of this internal policy is to act with individual responsibility in preserving the environment, minimizing environmental impacts through preservation actions such as reducing, reusing, and promoting the recycling of waste generated in the activity, in addition to optimizing the consumption of resources such as water and electricity.

To this end, a multidisciplinary internal committee called “*Gente Consciente*” (“Conscious People”) was set up with responsibility for implementing environmental policy, disseminating knowledge, and monitoring waste generation indicators and environmental actions at dialysis units.

### Solar Energy

HD clinics are major consumers of energy, which is used mainly to power dialysis machines and for air conditioning. In a British study that assessed the total carbon footprint per patient/year, energy consumption accounted for 21% of the total 3.8 tons of CO2Eq^11^.

In 2022, the institution received the installation of 515 solar panels (262 kWp) as part of an energy efficiency project for hospitals promoted by the local energy distributor, CELESC. The panels were installed on adjacent land and on the roof of the building that houses the institution’s administrative center and a hemodialysis unit serving approximately 150 patients. This initiative meets 76% of the building’s energy demand. In addition to significantly reducing carbon emissions, the annual energy cost savings are estimated at approximately R$ 136,724.64.

### Reuse of Reverse Osmosis Reject Water

The amount of water consumed for HD depends on several factors and can reach up to 500 liters per session. Of this total, approximately two-thirds are rejected by the reverse osmosis treatment system and discharged into the sewage system without any contact with patients’ blood^12^. The discarded water has a higher salt content, as it results from the deionization process, and may be used for various purposes. In some of the institution’s dialysis units, water that would otherwise be completely wasted is reused in the toilet flushing system, for garden irrigation, and general cleaning of the physical infrastructure, generating both environmental and financial savings.

### Material Recycling

In 2019, the “Transforming Recycling into Solidarity” project was implemented, which has continuously contributed to the provision of basic food baskets to socially vulnerable patients assisted by the institution.

The project aims to recycle 5- and 10-liter high-density polyethylene (HDPE) containers used for acidic or basic solutions during dialysis procedures, as well as the cardboard boxes used in their packaging. In 2023, an average of 839 kilograms of cardboard and 11,825 HDPE containers were generated per month at the Santa Catarina units, resulting in a total monthly revenue of R$ 3,799.54. The annual revenue of R$ 45,594.48 enabled the conversion of the resource into a total of 608 basic food baskets, donated monthly to 51 socially vulnerable patients undergoing dialysis treatment. As this amount is not enough to meet the needs of all patients in this condition, the institution also relies on food donations from the community and holds biannual bazaars for the sale of clothing and footwear, also donated by the community, thus promoting the circular economy. In 2023, over 5,500 items were sold, generating approximately R$ 72,000.00 in revenue.

#### Other sustainable actions

In addition to the aforementioned practices for reducing the environmental impact of operations, other actions are described in Chart 1. Among the main initiatives, the promotion of access to peritoneal dialysis as the first-line treatment option stands out, as it is associated with lower carbon emissions^13^.

**Chart 1:** Institutional actions with environmental, economic, or social impact

1) Dialysis treatment	*Treatment* – Encouraging peritoneal dialysis as a dialysis modality (urgent start program) – Peritoneal dialysis and incremental hemodialysis whenever indicated – Nutritionist consultations encouraging natural food consumption and reduction in the consumption of ultra-processed foods – Pharmacoeconomic actions – Safety protocols aimed at reducing the risk of complications *Resource management* – Solar energy – Reuse of water rejected by osmosis treatment – Material recycling – Dialyzers reuse – Reduced paper consumption (electronic medical records) *Laundry services* – Encouraging patients to bring their own blankets – Individual amenity kits with monthly sanitization (whenever possible) – Replacement of plastic bags for clothing transport with reusable material – Service provider with its own treatment station, enabling water reuse
2) Institutional resource savings	– Energy, paper, commuting (online meetings) – Collection of aluminum pull tabs to be exchanged for wheelchairs – Use of individual mugs by staff, reducing plastic cup consumption
3) Support for socially vulnerable patients	– Donation of basic food baskets purchased with funds from the sale of recyclable materials, second-hand clothing bazaars, and other community initiatives – Sorting blankets, clothes, and footwear for direct donation or for sale, with proceeds going toward the purchase of basic food baskets or allocated to other institutions

## Conclusion

The impact of kidney disease goes beyond caring for people on renal replacement therapy. The treatment of this condition not only entails economic costs but also has significant social and environmental repercussions. Such impacts can be mitigated through preventive actions and solutions such as those presented in this article. Committed to continuous improvement and aware of the need to implement many other initiatives, the institution has become a signatory to the United Nations Sustainable Development Goals.

By sharing initiatives such as those described in this article, we aim to engage the nephrology community and the more than 900 dialysis units in Brazil in adopting more sustainable practices.

## Data Availability

The complete dataset supporting the results of this study has been published within the article itself.
