# Supplementary material for: Green nephrology in practice: actions that promote environmental, social, and economic impact
Source: J Bras Nefrol. 2025 Jun 13;47(4):e20240206. doi: 10.1590/2175-8239-JBN-2024-0206en (PMC12172542; doi:10.1590/2175-8239-JBN-2024-0206en)
Supplement: Translation

# Translation: Nefrologia verde na prática: ações que promovem impacto ambiental, social e econômico

**DOI:** 10.1590/2175-8239-JBN-2024-0206pt

**Authors:** Fabiana Baggio Nerbass, Rosenilde Schulz, Maicon Aurélio de Vargas, Maycon Truppel Machado, Marcos Alexandre Vieira

**Affiliations:** 1 Fundação Pró-Rim, Joinville, SC, Brasil.

**Keywords:** Mudança Climática, Diálise, Reciclagem, Energia renovável

## Abstract

As mudanças climáticas afetam, e continuarão a afetar, a saúde global e renal de milhões de pessoas ao redor do mundo. Ao mesmo tempo em que os serviços de saúde devem estar preparados para o aumento da incidência e para as mudanças na distribuição das doenças renais, eles também contribuem substancialmente para o esgotamento dos recursos naturais e emissão de gases de efeito estufa. Dentre esses serviços, o setor de diálise destaca-se pelo impacto ambiental negativo, em razão de seu elevado consumo de água, energia e insumos, além da geração de resíduos. Assim, medidas que reduzam a utilização de recursos naturais no procedimento dialítico, bem como aquelas voltadas à diminuição do número de pacientes dependentes de diálise, como iniciativas de prevenção de doenças renais e de incentivo ao transplante renal, são de fundamental relevância. Além dos ganhos ambientais, ações internas focadas na implementação de práticas sustentáveis podem também gerar retornos econômicos. Neste artigo, nosso objetivo foi informar e contribuir para o conhecimento sobre experiências sustentáveis já implementadas em uma instituição de saúde filantrópica brasileira. Entre elas, descrevemos como a utilização de energia renovável, o reaproveitamento da água de rejeito da osmose reversa e a reciclagem de resíduos se revertem em redução de custos de operação e em assistência direta aos pacientes em situação de vulnerabilidade social.

## Introdução

De acordo com a Organização Mundial de Saúde, as alterações climáticas já contribuem diretamente para emergências humanitárias, e as populações que vivem em zonas tropicais, especialmente aquelas que habitam regiões com infraestrutura de saúde deficiente, serão as que continuarão sofrendo as maiores consequências^1^.

Em particular, as doenças renais e as alterações climáticas apresentam uma relação bidirecional: uma agrava a outra^2^. Em uma meta-análise com 82 estudos epidemiológicos, observou-se que, para cada 1ºC de aumento na temperatura, houve um acréscimo de 1% no risco de morbidade e de 3% na mortalidade por causas renais. Além disso, as ondas de calor aumentaram o risco de doenças renais de forma proporcional à sua intensidade^3^.

Por outro lado, o tratamento da doença renal crônica, especialmente por meio da hemodiálise (HD), apresenta uma das pegadas ecológicas mais elevadas em todo o espectro dos cuidados clínicos, devido ao seu alto consumo de energia, água e plástico descartável^4^, além da geração de efluentes moderadamente tóxicos^5^. A HD é o tratamento dialítico utilizado por aproximadamente 90% das pessoas dependentes de diálise no mundo^6^ e por mais de 95% das que fazem tratamento no Brasil, número que supera 150 mil indivíduos^7^.

Em uma publicação recente, foi anunciada a iniciativa “GREEN-K”: Evolução Ambiental Global em Nefrologia e Cuidados Renais (do inglês *Global Environmental Evolution in Nephrology and Kidney Care*), com a visão de “cuidados renais sustentáveis para um planeta saudável e rins saudáveis”. Entre outras propostas, a iniciativa defende a priorização de ações de promoção da saúde para prevenir e retardar o progresso da doença renal, melhorar o acesso ao transplante e atender à necessidade urgente de terapias dialíticas com menor impacto ambiental^2^. Uma pesquisa que contou com a participação de mais de 972 profissionais de saúde de 108 países, sobre os impactos das mudanças climáticas e ambientais na saúde e nos cuidados renais, revelou que, apesar da grande maioria afirmar preocupação com estas questões, apenas 26% relataram trabalhar em organizações com iniciativas sustentáveis. Esse percentual não variou de acordo com o nível de renda do país^8^.

Um apelo pela sustentabilidade na diálise no Brasil foi publicado em 2019, mas, como aponta o artigo, o tema ainda é pouco discutido no país. Os autores alertam para a necessidade de divulgar e incentivar a adoção da sustentabilidade nos centros de diálise de todo o país, promovendo a criação de uma consciência coletiva^9^.

Além dos ganhos ambientais, a implementação de práticas sustentáveis pode gerar benefícios tanto econômicos quanto socais. Neste artigo, nosso principal objetivo é a divulgação de ações sustentáveis bem-sucedidas de unidades de diálise de uma instituição filantrópica, como a utilização de energia renovável, o reaproveitamento da água de rejeito da osmose reversa e a reciclagem de materiais, que se revertem em redução de custos de operação e em assistência direta a pacientes em situação de vulnerabilidade social.

## Política de Gestão Ambiental Institucional

A instituição é uma entidade de administração privada, filantrópica e sem fins lucrativos, fundada em 1987, presente nos estados de Santa Catarina e Tocantins, onde mantém centros de diálise próprios e em parceria.

A política de gestão ambiental da instituição foi implantada logo após a divulgação da RDC nº 306, de 2004, que dispõe sobre o Regulamento Técnico para o Gerenciamento de Resíduos de Serviços de Saúde (PGRSS)^10^. O compromisso dessa política interna é atuar com responsabilidade individual na preservação do meio ambiente, minimizando os impactos ambientais por meio de ações de preservação como a redução, a reutilização e a promoção da reciclagem de resíduos gerados na atividade, além da otimização do consumo de recursos como água e energia elétrica.

Para isso, foi formada uma comissão interna multidisciplinar, intitulada “Gente Consciente”, com a responsabilidade de atuar sobre a política ambiental, disseminando conhecimento e monitorando indicadores de geração de resíduos e ações ambientais das unidades de diálise.

### Energia Solar

Clínicas de HD são grandes consumidoras de energia, utilizada especialmente para o funcionamento das máquinas dialisadoras e para a climatização. Em um estudo britânico que avaliou a pegada total de carbono por paciente/ano, o consumo de energia foi responsável por 21% do total de 3,8 toneladas de CO^2^Eq^11^.

Em 2022, a instituição recebeu a instalação de 515 placas solares (262 kWp), como parte de um projeto de eficiência energética para hospitais promovido pela distribuidora de energia local – CELESC. As placas foram instaladas em um terreno adjacente e na cobertura do edifício que abriga o centro administrativo da instituição e uma unidade de hemodiálise com cerca de 150 pacientes. Essa iniciativa supre 76% da demanda energética do edifício. Além de reduzir significativamente as emissões de carbono, a economia anual com energia é estimada em aproximadamente R$ 136.724,64.

### Reaproveitamento da Água de Rejeito da Osmose Reversa

A quantidade de água consumida para a HD depende de diversos fatores e pode chegar a 500 litros por sessão. Desse total, aproximadamente dois terços são rejeitados pelo sistema de tratamento por osmose reversa e descartados na rede de esgoto, sem qualquer contato com o sangue dos pacientes^12^. A água descartada é mais rica em sais, pois resulta do processo de deionização e pode ser utilizada para diversos propósitos. Em algumas unidades de diálise da instituição, a água que antes seria totalmente desperdiçada é utilizada no sistema de descarga dos banheiros, na irrigação dos jardins e na higienização geral da estrutura física, gerando economia ambiental e de recursos financeiros.

### Reciclagem de Materiais

Em 2019, foi implementado o projeto “Transformando a Reciclagem em Solidariedade”, o qual contribui continuamente para o benefício da cesta básica fornecida aos pacientes em situação de vulnerabilidade social atendidos pela instituição.

O projeto tem como objetivo a reciclagem de bombonas de 5 e 10 litros de polietileno de alta densidade (PEAD) de solução ácida ou básica utilizadas durante o procedimento dialítico, bem como das caixas de papelão que as acondicionam. No ano de 2023, foram gerados, em média, 839 quilos de papelão e 11.825 unidades de bombonas por mês nas unidades de Santa Catarina, totalizando uma receita mensal de R$ 3.799,54. A receita anual de R$ 45.594,48 viabilizou a conversão do recurso em um total de 608 cestas básicas, doadas mensalmente a 51 pacientes em tratamento dialítico em situação de vulnerabilidade social. Como esse montante não é suficiente para atender a todos os pacientes nessa condição, além da arrecadação de alimentos doados pela comunidade, a instituição promove, semestralmente, bazares para a venda de roupas e calçados também doados pela comunidade, estimulando, assim, a economia circular. Em 2023, mais de 5.500 peças foram comercializadas, totalizando uma arrecadação aproximada de R$ 72.000,00.

#### Outras ações sustentáveis

Além das práticas mencionadas anteriormente para a redução do impacto ambiental da operação, outras ações estão descritas no Quadro 1. Entre as principais, destaca-se a facilitação do acesso à diálise peritoneal como primeira modalidade de tratamento, uma opção associada a menor emissão de carbono^13^.

**Quadro 1:** Ações institucionais com impacto ambiental, econômico ou social

1) Tratamento dialítico	*Tratamento* – Incentivo à diálise peritoneal como modalidade dialítica (programa de início urgente) – Diálise peritoneal e hemodiálise incremental sempre que indicadas – Consultas com nutricionistas com incentivo à alimentação natural e redução do consumo de alimentos ultraprocessados – Ações de farmacoeconomia – Protocolos de segurança visando à diminuição do risco de complicações *Gerenciamento dos recursos* – Energia solar – Reaproveitamento da água rejeitada pelo tratamento da osmose – Reciclagem de materiais – Reuso de dialisadores – Redução da utilização de papéis (prontuários eletrônicos) *Lavanderia* – Incentivo aos pacientes para trazerem cobertores próprios – Kits de hotelaria individuais com higienização mensal (sempre que possível) – Substituição de sacos plásticos para transporte de roupas por material reutilizável – Prestador de serviço com estação de tratamento própria, que viabiliza a reutilização da água
2) Economia de recursos institucionais	– Energia, papel, deslocamentos (reuniões on-line) – Arrecadação de lacres de alumínio revertidos em cadeiras de rodas – Uso de canecas individuais pelos funcionários, diminuindo o consumo de copos plásticos
3) Assistência da vulnerabilidade social dos pacientes	– Doação de cestas básicas adquiridas com os recursos provenientes da venda de materiais para reciclagem, promoção de bazares de roupas usadas e outras ações na comunidade – Triagem de cobertores, roupas e calçados para doação direta ou para venda com geração de recursos revertidos na aquisição de cestas básicas ou destinados a outras instituições

## Conclusão

O impacto da doença renal transcende o atendimento às pessoas em terapia renal substitutiva. O tratamento dessa condição não apenas acarreta custos econômicos, mas também gera repercussões sociais e ambientais significativas. Tais impactos podem ser mitigados por meio de ações preventivas e de soluções como as apresentadas neste artigo. Comprometida com a melhoria contínua e ciente da necessidade de implementação de muitas outras iniciativas, a instituição tornou-se signatária dos Objetivos de Desenvolvimento Sustentável da Organização das Nações Unidas.

Ao compartilhar iniciativas como as descritas neste artigo, almejamos engajar a comunidade nefrológica e as mais de novecentas unidades de diálise no Brasil na adoção de práticas mais sustentáveis.

## Data Availability

Todo o conjunto de dados que dá suporte aos resultados deste estudo foi publicado no próprio artigo.
